# Multimorbidity prevalence in the general population: the role of obesity in chronic disease clustering

**DOI:** 10.1186/1471-2458-13-1161

**Published:** 2013-12-10

**Authors:** Calypse B Agborsangaya, Emmanuel Ngwakongnwi, Markus Lahtinen, Tim Cooke, Jeffrey A Johnson

**Affiliations:** 1 Department of Public Health Sciences, 2–040 Li Ka Shing Center for Health Research and Innovation, University of Alberta, Edmonton, Alberta T6G 2E1, Canada; 2 Health Quality Council of Alberta, Calgary, Alberta, Canada

**Keywords:** Multimorbidity, Obesity, Chronic diseases, Health outcome

## Abstract

**Abstract:**

**Background**

The role of obesity in the prevalence and clustering of multimorbidity, the occurrence of two or more chronic conditions, is understudied. We estimated the prevalence of multimorbidity by obesity status, and the interaction of obesity with other predictors of multimorbidity.

**Methods**

Data from adult respondents (18 years and over) to the Health Quality Council of Alberta 2012 Patient Experience Survey were analyzed. Multivariable regression models were fitted to test for associations.

**Results**

The survey sample included 4803 respondents; 55.8% were female and the mean age was 47.8 years (SD, 17.1). The majority (62.0%) of respondents reported having at least one chronic condition. The prevalence of multimorbidity, including obesity, was 36.0% (95% CI, 34.8 – 37.3). The prevalence of obesity alone was 28.1% (95% CI 26.6 – 29.5). Having obesity was associated with more than double the odds of multimorbidity (odds ratio = 2.2, 95% CI 1.9 – 2.7) compared to non-obese.

**Conclusions**

The prevalence of multimorbidity in the general population is high, but even higher in obese than non-obese persons. These findings may be relevant for surveillance, prevention and management strategies for multimorbidity.

## Background

Multimorbidity, the concurrent occurrence of two or more chronic conditions [1], is an emerging issue in public health agenda because of its increasing prevalence [2,3], impact on individual health status, and the economic impact on the health care system. Persons with multimorbidity are thought to be at increased risk of receiving sub-optimal care [4,5], more frequent and longer hospitalizations, higher health care costs and increased use of polypharmacy with the potential for adverse drug effects [6,7].

Estimates of the prevalence of multimorbidity vary from 17% to over 90% [1,8-10]. This wide variation is due to dissimilar definitions and data sources, reflecting differences in demographic characteristics and types of chronic conditions [2,11]. Another important difference in previous studies is the inclusion of chronic conditions such as obesity and high cholesterol, typically considered as risk factors for other chronic conditions. Obesity, for instance, is considered an entrance port to multimorbidity [12] and an important risk factor for future morbidity. Its inclusion in studies on multimorbidity is thought to be vital [13,14]. However, of 39 multimorbidity indices included in a systematic review [11], only five studies included obesity in the count of chronic conditions. Overall, the impact of obesity on multimorbid disease clustering in chronic diseases is largely understudied.

The aim of this study was firstly to evaluate the prevalence of multimorbidity based on a list of 16 chronic conditions. Second, the clustering of multimorbidity by obesity status and other predictors was evaluated in a sample of the general adult population of Alberta, Canada.

We hypothesized that multimorbidity is more common in the obese than non-obese population.

## Methods

Data were available from the Health Quality Council of Alberta (HQCA) 2012 Patient Experience Survey [15]. The survey evaluated a representative sample of the general adult population (≥ 18 years of age) in Alberta on their experience and satisfaction with the quality of health services received in the past twelve months. The survey instrument, a telephone-based questionnaire, was administered by Random-Digit Dialing (RDD) approach to ensure that households in each of five health zones had an equal chance of being contacted. Data were collected in the spring of 2012. Details of the survey are available [16]. Sampling weights were derived based on the age and sex distribution of adult Albertans to account for the stratified sampling approach, across all five health zones of Alberta [16].

In addition to socio-demographic information (i.e., age, sex, education and annual household income), respondents were asked about the presence of chronic conditions by answering the question “*Do you have any of the following chronic conditions or diseases?”;* diabetes, chronic obstructive pulmonary disorder, asthma, hypertension, high cholesterol, sleep apnea, congestive heart failure, depression or anxiety, chronic pain, arthritis, heart disease, stroke (or related conditions), cancer, bowel disease and kidney disease.

Respondents were also asked to report their weight and height. Body mass index (BMI) was calculated (BMI = Weight/Height^2^) for each respondent and adjusted for self-report bias using sex-specific adjustment equations [17]. Obesity was defined as a BMI ≥ 30 kg/m^2^. This study, therefore, considered a total of 16 chronic conditions. We were primarily interested in multimorbidity, which was defined as the concurrent occurrence of two or more of these chronic conditions in the same individual. However, for data presented by obesity status, obesity itself was not included as a chronic condition in the definition of multimorbidity.

### Data analysis

The prevalence of multimorbidity was estimated in relation to age, sex, household income and educational level. Educational level was collapsed into three categories; high school (at most high school education), college (more than high school education, including completion of college education), and university (at least university degree). Prevalence measures were direct- standardized to the 2006 age and sex distribution of the Alberta population (i.e., based on 2006 Canadian Census). The prevalence of multimorbidity with/without obesity as a chronic condition was reported for comparison purposes.

A multivariable logistic regression model was fitted to evaluate the association between obesity and multimorbidity, adjusting for other covariates. Because obesity status was added to the model as a predictor, our multimorbidity definition for the regression analysis did not include obesity. To examine differential effects of obesity on the occurrence of multimorbidity by socio-demographic characteristics, we tested for interactions between obesity and each of the socio-economic covariates (i.e., age, sex, education, household income), considering p-value of <0.10 to indicate an important interaction. Population sampling weights were applied in all regression models. All statistical analyses and data management were performed using STATA V11 package. The Health Research Ethics Board (HREB) at the University of Alberta approved the data collection protocols and survey instruments.

## Results

### Study sample

The survey sample included 4803 respondents, 55.8% were female and the mean age was 47.8 years (SD, 17.1). The socio-demographic characteristics of the respondents are presented in Table 1. The majority of the respondents had a household income of at least 60,000 dollars (62.5%). Including obesity as a chronic condition, 2979 (62.0%) respondents reported having at least one chronic condition, with an average of 2.6 (1.8) chronic conditions. The prevalence of obesity was 28.1% (95% CI 26.6 – 29.5). Obese persons were significantly older than non-obese population (50.4 versus 46.7, p < 0.0001).

**Table 1:** Socio-demographic characteristics of the respondents to the 2012 Health Quality Council of Alberta’s Patient Experience Survey

**Characteristic**	***N (4803)***	**%**
*Sex*		
Females	2680	55.8
*Age (years)*		
18 – 24	529	11.0
25 – 44	1619	33.7
45 – 64	1842	38.4
65+	813	16.9
*Household income (CAD)*		
≥ 100,000	1504	36
60,000 – 99,999	1105	26.5
30,000 – 59,999	935	22.4
< 30,000	634	15.2
*Education*		
University degree	1218	25.5
Some post-secondary/college diploma	1916	40.0
Secondary or less	1652	34.5
^*^Health Zone		
South	890	18.5
Calgary	1095	22.8
Central	862	18.0
Edmonton	1078	22.4
North	878	18.3

^*^Alberta health zones.

### Prevalence of multimorbidity

The age and sex standardized prevalence of multimorbidity was 36.1% (95% CI 34.7 – 37.3) in the surveyed general population, and 60.5% (95% CI 58.5 – 62.6) among respondents who reported at least one chronic condition. When obesity was excluded as a chronic condition, the prevalence of multimorbidity was 30.9% (95% CI 29.5 – 32.4).

The age-standardized prevalence of multimorbidity was similar in females (36.5%, 95% CI 34.8 – 38.1) and males (35.5%, 95% CI 33.6 – 37.5). Across different age groups, the prevalence increased from 12.5% (9.7 – 15.4) in the youngest age group (18–24 years) to 63.8% (95% CI 60.5 – 67.1) in the oldest (≥ 65 years). When categorized by household income levels, the prevalence of multimorbidity increased with decreasing household income, from 29.7% (95% CI 27.2 – 32.4) in the highest income group (≥ $100,000) to 53.7% (95% CI 49.5 – 58.0) in the lowest income group (< $30,000) (Table 2).

**Table 2:** Prevalence of multimorbidity across socio-demographic characteristics

***Variables***	***Mean number of chronic conditions (SD)***	***Multimorbidity prevalence (%)***	
		**Crude**	**Adjusted (95% CI)**
^*1*^*Sex*			
Males	1.6 (1.8)	38.2	35.5 (33.6 – 37.5)
Females	1.6 (1.9)	39.6	36.5 (34.8 – 38.2)
^*2*^*Age Group*			
18 – 24	0.5 (1.0)	12.5	12.6 (9.7 – 15.4)
25 – 44	1.0 (1.3)	24.0	24.1 (21.9 – 26.2)
45 – 64	2.1 (2.0)	48.2	48.2 (45.9 – 50.4)
65+	2.7 (2.2)	64.1	63.8 (60.5 – 67.1)
^*3*^*Income*			
> = $100,000	1.1 (1.5)	28.3	29.8 (27.2 – 32.3)
$60,000 – 99,000	1.4 (1.6)	36.2	35.3 (32.6 – 38.0)
$30,000 – 59,000	1.9 (2.0)	44.3	37.8 (34.7 – 40.9)
< $30,000	2.7 (2.4)	61.4	53.7 (49.5 – 58.0)
*Education*			
University degree	1.2 (1.6)	31.5	29.9 (27.3 – 32.5)
Some post-secondary/college diploma	1.5 (1.8)	37.2	35.8 (33.7 – 37.9)
Secondary or less	2.0 (2.1)	45.9	41.7 (39.3 – 44.1)
^*3*^*Health Zones*			
South	1.7 (1.9)	41.3	37.1 (34.0 – 40.1)
Calgary	1.4 (1.8)	33.7	31.0 (29.4 – 34.6)
Central	1.8 (2.0)	41.9	37.3 (34.1 -40.4)
Edmonton	1.6 (1.9)	39.4	37.8 (34.9 – 40.3)
North	1.6 (1.8)	38.7	37.3 (34.3 – 40.3)

^1^Age-standardized. ^2^Sex-standardized. ^3^Age- and Sex-standardized. Multimorbidity is defined as the presence of two or more chronic conditions (Including obesity as a chronic condition).

**Figure 1:**
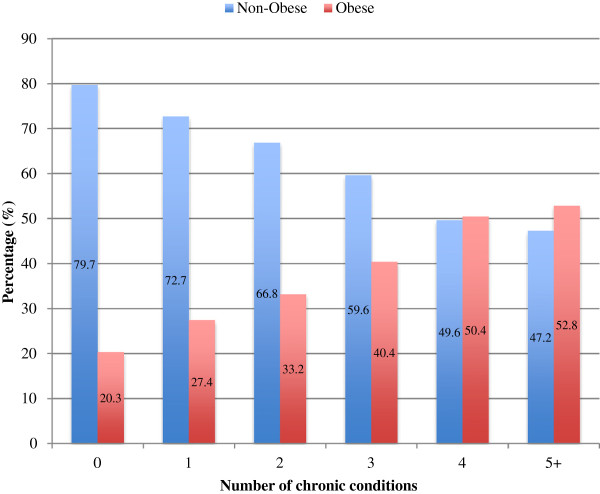
The prevalence of obesity increases with increasing number of chronic conditions (Obesity was not included in the number of chronic conditions).

**Figure 2:**
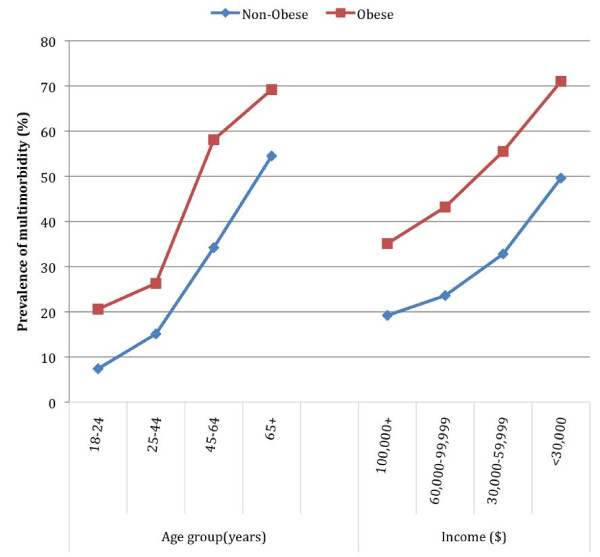
The standardized age- and income-specific prevalence of multimorbidity in the obese and non-obese population (Obesity was not included in the definition of multimorbidity).

The mean number of chronic conditions was significantly higher in the obese population (1.9, SD 2.0) than in the non-obese population (1.0, SD 1.5), p < 0.0001. Multimorbidity was significantly more common in obese persons (42.7%, 95% CI 40.2 – 45.3) compared to non-obese persons (25.9%, 95% CI 24.4 – 27.3), p < 0.0001. Obesity prevalence increased with increasing number of chronic conditions (Figure 1), being lowest among those with no chronic condition (20.3%, 95% CI 18.3 – 22.2) and highest in those with 5 or more chronic conditions (52.8%, CI 46.4 – 59.2). Likewise, the age- and income-specific prevalence of multimorbidity was higher in the obese population than in the non-obese population for all age and household income groups (Figure 2).

### Multimorbidity combinations

The most common chronic condition in persons with obesity was high blood pressure (19.3%), while arthritis (21.9%) was most common in the non-obese population. The most common disease pairs and triads in the obese population were chronic pain – arthritis (11.2%) and high blood pressure – high cholesterol – arthritis (11.0%), respectively. In the non-obese population, they were chronic pain – arthritis (12.6%) and anxiety/depression – chronic pain – arthritis (8.1%).

### Multimorbidity correlates

In the multivariable analysis, socio-demographic predictors of multimorbidity were age (OR = 12.6, 95% CI 8.3 – 19.2, for lowest versus highest), educational level (OR = 1.5, 95% CI 1.2 – 1.9, for lowest versus highest), and household income (OR = 2.8, 95% CI 2.2 – 3.7, for highest versus lowest). Being obese was associated with more than two-fold greater odds of having multimorbidity (OR = 2.2, 95% CI 1.9 – 2.6) in the general population (Table 3). No statistically significant interactions between obesity and any of the socio-economic characteristics were found (p > 0.3 for all terms).

**Table 3:** **Multivariable analysis of the association between characteristics and multimorbidity**^**1**^

***Variable***	***Odds ratio***	***95% Confidence intervals***
*Obesity status*		
Non-obese	1.0 (ref)	
Obese	*2.2*	*1.9 – 2.7*
*Sex*		
Males	1.0 (ref)	
Females	1.1	0.9 – 1.2
*Age Group*		
18 – 24	1.0	
25 – 44	2.3	1.5 – 3.4
45 – 64	7.2	4.8 – 10.7
65+	12.6	8.3 – 19.2
Income		
> = $100,000	1.0 (ref)	
$60,000 – 99,999	*1.3*	*1.1 – 1.6*
$30,000 – 59,999	*1.6*	*1.3 – 2.1*
< $30,000	*2.9*	*2.2 – 3.7*
*Education*		
University degree	1.0 (ref)	
Some post-secondary/college diploma	1.1	0.9 – 1.4
Secondary or less	*1.5*	*1.2 – 1.8*

^**1**^All variables are adjusted for each other. Multimorbidity is defined as the presence of two or more chronic conditions (Including Obesity as a chronic condition).

## Discussion

In the present study, based on a sample of the general adult population of Alberta, the prevalence of multimorbidity, including obesity, was 36%; minus obesity, it was 31%. Cardiovascular comorbidities tended to be more commonly reported amongst the obese and non-obese populations alike. Consistent with our hypothesis, persons with obesity were more than twice as likely to report having multimorbidity than the non-obese population. Moreover, the prevalence of multimorbidity was consistently higher among obese persons compared to non-obese persons for the same age and household income levels.

These findings are consistent with previous indications that obesity is an important risk factor for morbidity and thought to be an entrance port to multimorbidity [12,18]. In a population-based registry study in Sweden, the authors observed that obesity was associated with a three-fold increased likelihood of having multimorbidity [18]. In another study among HIV-infected patients, the prevalence of multimorbidity tended to increase progressively with increasing BMI categories [19].

The inclusion of obesity in multimorbidity indices has been largely contested [14,20], likely as an extension of the controversy of considering obesity as a chronic condition. Given that the prevalence of obesity is increasing and that it is associated with increased risk of adverse health states, including all-cause mortality [21], there is need for its inclusion in multimorbidity indices. An alternate approach might be to only consider morbid obesity (i.e., BMI > 40) [20], which, as a subset of obesity, would result in a lower prevalence of multimorbidity. This study showed that obesity prevalence positively correlates with multimorbidity, with obesity being more common in populations with higher number of chronic conditions. In fact, the increasing prevalence of multimorbidity with increasing age and lower income tended to be higher in obese than non-obese persons. The observed differences in our study make a case for the inclusion of obesity, otherwise severe obesity, as a chronic condition in multimorbidity indices, in the same way that uncomplicated hypertension is included [14].

This study has several limitations. The cross-sectional nature of the data prevents the examination of the temporality of the associations that have been evaluated. Despite the inclusion of up to 16 chronic conditions, numerous conditions prevalent in the study population were not included. Considering that the prevalence of multimorbidity depends on the number and type of chronic conditions in the multimorbidity index [2], the reported prevalence in this study may significantly change with increasing number of chronic conditions. It is possible that surveyed patients with none of the listed morbidities may have other unlisted chronic conditions. Consideration should therefore be given in the interpretation of these findings. Moreover, the data was based on self-reports. Self-reported chronic disease status is subject to self-declaration bias due to under-reporting of diagnosis or forgetfulness [22,23]. Some individuals who report having multimorbidity may essentially be reporting a single chronic condition and its symptom, e.g. arthritis and chronic pain. This may lead to over-estimation of the true prevalence of multimorbidity. A further limitation of this study is the absence of an indicator of disease severity. The allocation of an equal weight to all chronic conditions fundamentally assumes that the listed chronic conditions are the same. A further step may be to incorporate a severity weight to the chronic conditions.

Important strengths of this study include the fact that the chronic conditions included are common conditions the general Canadian population, and in other developed countries. The findings may therefore be of public health significance. Moreover, the modest number of chronic conditions considered in this study includes the core chronic conditions recommended for inclusion in multimorbidity indices [11]. Important chronic conditions such as obesity and high cholesterol that may contribute to morbidity of other adverse health states were included in the definition of multimorbidity.

## Conclusion

This study evaluates the prevalence of multimorbidity and provides an in-depth analysis of the impact of obesity in multimorbidity clustering. Obesity was an important independent predictor of the occurrence of multimorbidity. These findings highlight the importance of including obesity in multimorbidity indices, and may be vital for public health surveillance.

## Competing interests

The authors declare that they have no competing interests.

## Authors’ contributions

ACB: conception and design, statistical analysis and interpretation of data, drafting. manuscript, revision of manuscript. EN: data acquisition, interpretation of data, critical revision of manuscript. ML: data acquisition, survey instrument and design, critical revision of manuscript. TC: data acquisition, survey instrument and design, critical revision of manuscript. JAJ: conception and design, data acquisition and interpretation of data, critical revision of manuscript. All authors’ read and approved the final manuscript.

## Pre-publication history

The pre-publication history for this paper can be accessed here:

http://www.biomedcentral.com/1471-2458/13/1161/prepub
